# An Apatite-Group Praseodymium Carbonate Fluoroxybritholite: Hydrothermal Synthesis, Crystal Structure, and Implications for Natural and Synthetic Britholites

**DOI:** 10.1021/acs.inorgchem.4c01490

**Published:** 2024-06-13

**Authors:** Michael Anenburg, Taras L. Panikorovskii, Eleanor S. Jennings, Roman Yu. Shendrik, Andrey A. Antonov, Veronika Gavrilenko

**Affiliations:** † Research School of Earth Sciences, Australian National University, Canberra 2600, Australia; ‡ Laboratory of Nature-Inspired Technologies and Environmental Safety of the Arctic, Kola Science Centre, Russian Academy of Sciences, Apatity 184200, Russia; § School of Natural Sciences, Birkbeck, University of London, London WC1E 7HX, United Kingdom; ∥ Vinogradov Institute of Geochemistry, Siberian Branch, Russian Academy of Sciences, Irkutsk 664033, Russia

## Abstract

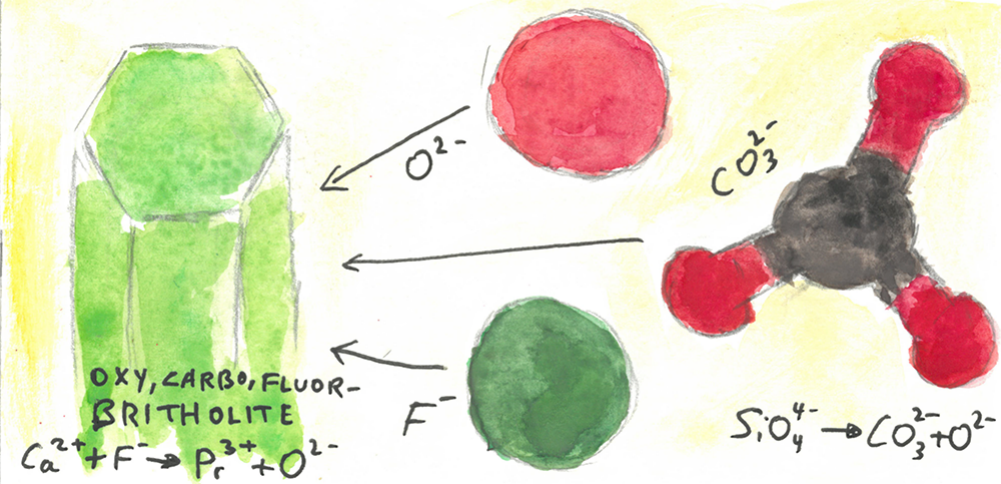

Britholites are the lanthanide–silica-rich end-members of the apatite group, commonly studied for their optical properties. Here, we show ∼50–100 μm single crystals synthesized hydrothermally at 650–500 °C and 500–300 MPa composed of a solid solution between Ca_2_Pr_3_(SiO_4_)_3_F–fluorbritholite and CaPr_4_(SiO_4_)_3_O–oxybritholite, with a significant carbonate component substitution, via C^4+^ replacing Si^4+^. Single-crystal X-ray diffraction and density functional theory computations show that a planar carbonate group occupies the face of a now-vacant silica tetrahedron. This modifies Pr–O bond lengths, diversifying lanthanide optical emission wavelengths. Our britholite was synthesized in geologically reasonable conditions and compositions, suggesting that carbonated oxybritholites could exist as yet-unrecognized natural minerals.

## Abstract 2

We prepared praseodymium fluor-britholite (ideally Ca_2_Pr_3_(SiO_4_)F) in which we show that excess Pr can be accommodated by replacement of fluorine by oxygen. We also show replacement of tetrahedral silicate groups by planar carbonate groups, with an excess oxygen pushed away from the carbonate. These structural modifications lead to wider optical emission spectra, and may explain enigmatic compositions of natural britholites.

## Introduction

Oxybritholite—Ca*Ln*_4_(SiO_4_)_3_O—and other lanthanide-bearing oxyapatites are used in applications such as optics,^1−9^ biomedical materials,^1,3,10−13^ nuclear waste immobilization,^14−20^ and solid fuel cells,^21−23^ where “*Ln*” refers to the lanthanides La–Lu and Y (commonly known as the rare earth elements: REE). Synthesis methods include hydrothermal growth,^10,24−27^ solid-state sintering,^4,14,15,21−23,28−34^ occasionally with preliminary treatments such as sol–gel synthesis,^1^ or microwave radiation.^10^ These methods commonly result in crystals only a few micrometres large, nanocrystals, or long but thin needles about 1–2 μm thick.^1−3,9−11,15−17,25,26,35,36^ This crystal morphology may not be suitable for all applications. Additionally, these methods result in either endmember britholite—Ca_2_*Ln*_3_(SiO_4_)_3_OH—or oxybritholite, with no intermediate compositions that may be useful in certain applications. Flux melting methods result in crystals 50 μm and larger, but the composition is limited to solutions of oxybritholite or fluorbritholite—Ca_2_*Ln*_3_(SiO_4_)_3_F—as these are done at high temperature in air, volatilizing all hydrogen and presumably all carbonate.^7,24,37−40^

Here, we show britholite grown in a high-pressure hydrothermal apparatus and characterized using a variety of microanalytical, optical, computational, and structural methods. We use the term “britholite” to refer to our synthetic compound which consists of the carbonate-bearing solid solution between fluorbritholite and oxybritholite, noting that the britholite component *sensu stricto* contains a hydroxyl component which was a negligible component in our materials. We discuss potential applications and ways to control the britholite composition. Finally, we discuss our findings in the context of naturally occurring apatite- and britholite-group minerals.

## Methods

The crystals described herein were originally synthesized for an earlier study on REE mobility in geological hydrothermal settings (run D2182).^41^ The lanthanide of choice for that study was Pr, because it was a single “average” representative of the light lanthanide series (La to Nd), making it easier to synthesize and subsequently analyze. One of the products of this experiment was britholite, which upon further preliminary investigation showed unusual chemical composition and potential for novel applications in chemistry, motivating the current study.

### Experimental Synthesis

A silver capsule was filled with powder layers, according to Table 1. The layered approach was chosen to initially form distinct zones within the capsule where different materials form and to explore the mobility of the various chemical components between zones at high temperature and pressure conditions. A 36.2 mg Ca–Cl–carbonate solution (prepared by fully neutralizing a 1 M HCl solution with CaCO_3_) was added to the solid starting materials. We note that not all starting materials are necessary for britholite synthesis. For example, the “SG” rock component (Table 1) can probably be replaced with pure silica, the fluorapatite layer can be removed completely, and MgCO_3_ can be replaced with CaCO_3_. The silver capsule was subsequently swaged and cold-welded according to previously described methods.^42^ We used an end-loaded piston cylinder apparatus with a 3/4 in. (19 mm) assembly size. The silver capsule was placed in a cylindrical MgO rod with a 10 mm graphite tube heater surrounded by a 19 mm talc sleeve and Teflon foil. The assembly was pressurized to 500 MPa and heated to 650 °C over 4 h. It was then held for 1 day at 650 °C and cooled to 500 °C over 4 days, while the pressure was held at 500 MPa for 2 days and decompressed to 300 MPa over 3 days (i.e., a total of 5 days for the experiment). The experiment was ended by quenching to room temperature, and the capsule was removed from the assembly and sectioned into two halves, which were mounted in epoxy resin for further analysis. No special safety precautions were required for the starting materials, as none were hazardous. Acids were treated by using appropriate protocols for dilute solutions. High-pressure experiments were conducted in accordance with well-established risk assessments and mitigations.

**Table 1:** Chemical Components Used in the Starting Mix for the Britholite Synthesis Experiment, Added as Layers to a Silver Capsule

compound	mass (mg)	purpose
Re metal	17.4	oxygen fugacity buffer
SG^b^	50.8	“rock”
MgCO_3_	11.4	low-*T* carbonate source
CaF_2_	2.1	fluoride source
Pr_6_O_11_	12.2	lanthanide layer
fluorapatite^a^	24.8	phosphate source
CaCO_3_	11.8	high-*T* carbonate source
SG^b^	9.2	“rock”
Re metal	11.7	oxygen fugacity buffer

a Introduced as stoichiometric mix of Ca_3_(PO_4_)_2_ and CaF_2_.

b Sintered mix of SiO_2_: 68.91%, Al_2_O_3_: 15.99%, Fe_2_O_3_: 6.21%, CaO: 3.15%, Na_2_O%: 2.36, K_2_O: 3.38%.

### X-ray Diffraction

For a single crystal X-ray diffraction (SCXRD) experiment, a britholite crystal was extracted from the surface of the polished silver capsule, fixed on a micromount, and placed onto an Agilent Technologies Xcalibur Eos diffractometer. The X-ray diffraction data were measured at 293 K using monochromated Mo Kα radiation. A hemisphere of three-dimensional data was collected with frame widths of 1° in ω and with a 20 s exposure time. The unit-cell parameters were refined by least-squares techniques using 462 reflections in the 2θ range of 7.62–55.00. For other details of data collection and structure refinement, see Table 2.

**Table 2:** Crystal Data and Structure Refinement for Our Synthetic Britholite

temperature (K)	293(2)
crystal system	hexagonal
space group	*P*6_3_/*m*
*a* = *b* (Å)	9.5588(4)
*c* (Å)	7.0097(4)
α = β (deg)	90
γ (deg)	120
volume (Å^3^)	554.67(6)
*Z*	2
ρ_calc_ (g/cm^3^)	5.037
μ (mm^–1^)	16.057
F(000)	761.0
crystal size (mm^3^)	0.17 × 0.15 × 0.07
radiation	Mo Kα (λ = 0.71073)
2Θ range for data collection/°	7.618 to 54.962
index ranges	–12 ≤ *h* ≤ 8, −8 ≤ *k* ≤ 12, −8 ≤ *l* ≤ 9
reflections collected	2389
independent reflections	462 [*R*_*i*nt_ = 0.0463, *R*_sigma_ = 0.0271]
data/restraints/parameters	462/10/43
goodness-of-fit on F^2^	1.194
final *R* indexes [I ≥ 2σ (I)]	*R*_1_ = 0.0283, w*R*_2_ = 0.0722
final *R* indexes [all data]	*R*_1_ = 0.0294, w*R*_2_ = 0.0728
largest diff. peak/hole (e Å^–3^)	1.60/–0.98

The crystal structure of our britholite refined in the *P*6_3_/*m* space group to *R* = 0.028 by means of the *SHELXL* program^43^ incorporated into Olex2 program.^44^ Empirical absorption correction was applied in the CrysAlisPro program complex using spherical harmonics, implemented in the SCALE3 ABSPACK scaling algorithm. Atom labels given according to actual classification of apatite supergroup of minerals.^45^ Visualizations of the crystal structure were performed in the VESTA program.^46^

A powder XRD spectrum was simulated using the algorithms of RIETAN-FP^47^ incorporated in the VESTA program.^46^ The powder XRD pattern was calculated with wavelengths α1 = 1.54059 Å and α2 = 1.54432 Å and Bragg–Brentano geometry in the 2θ range of 0–120°, and full width at half-maximum (fwhm) set 0.0745.

### Chemical analysis

Quantitative chemical composition of britholite (excluding C and H) was obtained using a field-emission JEOL 8530F Plus electron probe microanalyzer (EPMA) at the Centre for Advanced Microscopy, Australian National University, employing wavelength dispersive spectroscopy (WDS). Full spectrometer scans were conducted on the following diffracting crystals: layered dispersive element (LDE1), large thallium acid phthalate (TAP), large pentaerythriol (PET), and large lithium fluoride (LIF). Operating conditions during the scan were 15 kV, 100 nA, and a beam diameter of 40 μm. Dwell time was 700 ms with a 20 μm step size, scanning from ∼70 to ∼250 mm on each spectrometer. Once the elements of interest were identified, 20 spots were measured on different crystals using a beam current of 10 nA and diameter of 20 μm in order to minimize F migration on random orientations.^48−50^ We used the following X-ray lines: F Kα, Si Kα, Ca Kα, Pr Lα, and Fe Kβ (as the Fe Kα background positions had interference from Pr Lγ). All elements were analyzed for 20 s on peak and 10 s on each background. Reference materials were fluorite (CaF_2_) for F, diopside (CaMgSi_2_O_6_) for Si and Ca, hematite (Fe_2_O_3_) for Fe, and Pr-pentaphosphate (PrP_5_O_14_) for Pr. All reference materials were sourced from Astimex.

### Infrared Spectroscopy

Fourier transform infrared spectroscopy attenuated total reflectance (FTIR-ATR) was conducted by using a germanium crystal mounted on a Bruker A590 microscope with a Bruker IFS28 spectrometer and HgCdTe (MCT) detector. Spot size was ∼37.5 μm^2^, and contact force was 4 N on polished sections through several single crystals of britholite, which were thoroughly cleaned with organic solvents and deionized water to remove any potential contamination or surface residues. Background and ATR corrections using a refractive index of 1.79 were applied using the OPUS software. An atmospheric correction was applied to remove the infrared signal of gaseous CO_2_ at ∼2340 cm^–1^.

### Raman Spectroscopy

Raman spectral measurements were performed using a WITec alpha300R confocal Raman spectroscopic system coupled with a frequency-doubled 532 nm Nd:YAG laser at room temperature, calibrated on crystalline silicon. The spectra were recorded with a diffraction grating of 1800 lines per millimeter and a spectral resolution of 3 cm^–1^. The laser beam had an output power of 12 mW, and the focal spot diameter sample was between 5 and 10 μm. The backscattered Raman signal was collected by using a Zeiss 50×/NA 0.55 objective in a UHTS300 spectrometer equipped with a Peltier-cooled, front-illuminated CCD camera. Spectral scan durations were 30 s, with signals averaging over 5 scans. Raman spectra were processed using the ArDi web application.^51^

### Photoluminescence

Photoluminescence spectra were measured under 266 nm excitation by using a laser diode. Luminescence was registered using an Acton SP-2–500 spectrograph. The photoluminescence spectrum under 447 nm excitation was measured using an MDR2 grating monochromator and a H6780–04 Hamamatsu photomodule operating in counting mode with a spectral slit width of approximately 0.1 nm. The excitation was performed by using a 447 nm diode laser (837 mW).

### Ab Initio Calculations

Geometry optimization was performed using the Broyden–Fletcher–Goldfrab–Shanno (BFGS) iteration technique and delocalized internals minimizer. Self-consistency procedures conducted by dint of plane wave basis sets, Ceperley–Alder and Perdew–Zunger (CA-PZ) exchange-correlation functional, and on-the-fly generated (OTFG) norm-conversing pseudopotentials method within the local density approximation (LDA) formalism. The system was treated by ensemble density functional (EDFT) method: a self-consistent all-bands wave function search was performed, which for metals is followed by the self-consistent updating of occupancies. Relativistic effects were considered with a zeroth-order regular approximation (ZORA) to the Dirac equation. This approach was implemented in the CASTEP package.

The SCXRD-obtained crystal structure model was converted into the *Pm* space group, with half of C sites at 0.05 occupancy and half of Si sites at 0.95 occupancy were changed into fully occupied C and Si sites. Two half-populated *X*1 sites with a *X*1-*X*1 distance of 0.98 Å were changed to a fully occupied *X*1 site in the middle position. To account for charge balance constraints, the *M*1 site populated by Ca^2+^ whereas *M*2 was populated by Pr^3+^.

Energy cutoff (789.1 eV/atom), self-consistent field (SCF) tolerance (2 × 10^–6^ eV/atom), and Monkhorst–Pack mesh dimension (2 × 2 × 2) values were selected to obtain the desired convergence: 2 × 10^–4^ eV/atom for energy, 3 × 10^–1^ eV/Å for maximum force, 1 GPa for maximum stress, and 0.2 Å for maximum displacement.

## Results

**Figure 1:**
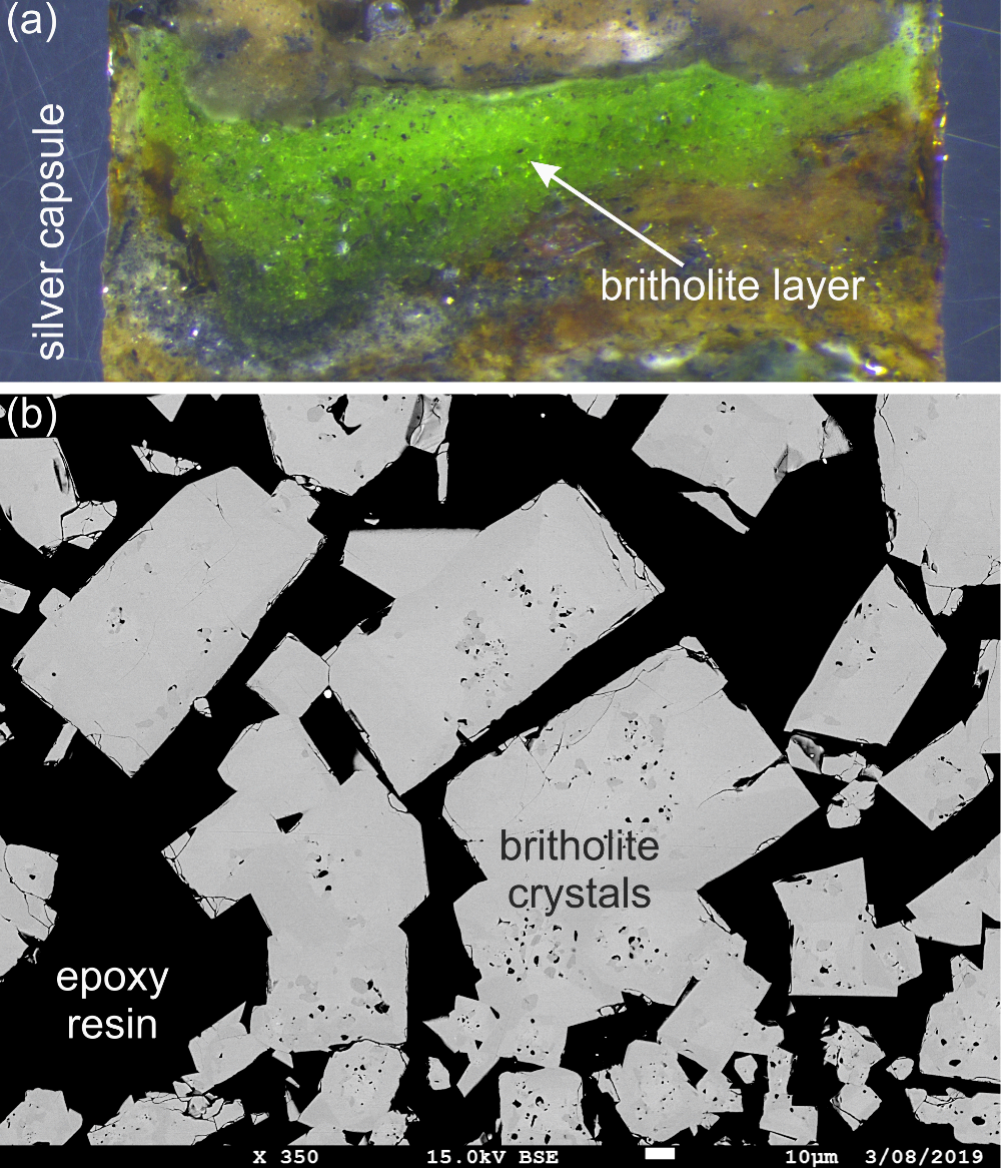
(a) Reflected light image of a sectioned capsule. Gray surface on the edges is silver, bright green is britholite, and the various browns are byproduct silicate material. Width of the image is about 6 mm. (b) Backscattered electron image of britholite crystals. Note the minor zoning that follows crystal growth patterns. Black is an epoxy resin.

The initial Pr_6_O_11_-bearing layer recrystallized to euhedral to subhedral crystals of britholite, some of which exhibit hexagonal crystal form characteristic for apatite supergroup minerals. The crystals were erroneously identified as cerite in a previous study due to nonstoichiometry.^41^ The crystals appear green as expected from a Pr^3+^-rich material, due to the utilization of a Re oxygen buffer,^52^ preventing the formation of Pr^4+^.^53^ Individual crystals can be easily seen in a simple reflected-light optical image (Figure 1a).

### Chemical Composition

**Figure 2:**
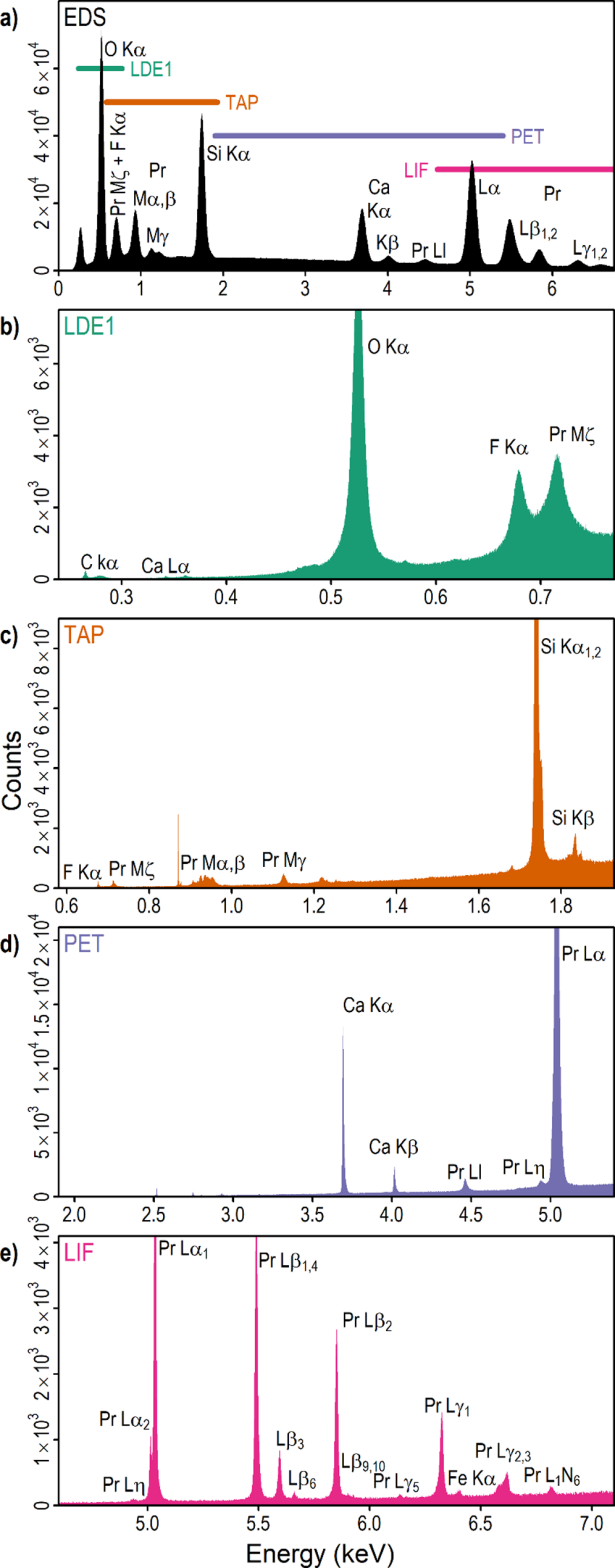
(a) Representative EDS spectrum of a britholite crystal, showing overlap of F and Pr. Energy range of each WDS analyzing crystal is shown by the colored horizontal bands. (b, e) WDS scans of britholite using the different analyzing crystals. Only first order peaks are annotated, despite the occurrence of second and higher order peaks (identified by lower intensity and atypical sharp resolution).

Preliminary analysis of britholite grains by EDS revealed that their mass composition is 20.33% SiO_2_, 8.20% CaO, and 67.63% Pr_2_O_3_ for a total of 96.17%, consistent with (Ca_1.30_Pr_3.64_)_∑4.94_Si_3.00_*X*, showing nonstoichiometry (i.e., Pr > 3 atoms per formula unit—apfu) and raising the question of which charge-balancing ions occupy the *X*-site. Furthermore, the cation sum of 4.94 also indicated significant *M*-site vacancies. To further confirm the chemical composition, we first conducted a full WDS spectrometer scan to identify all major and minor elements, which in comparison to the EDS spectrum, (1) identified the presence of F, whose Kα line is indistinguishable from the Pr Mζ line (compare Figure 2a,b), and (2) revealed minor Fe whose Kα line was obscured by the various Pr Lγ lines (Figure 2). Additionally, the WDS scan demonstrated the overall purity of the material, which was surprising given the multitude of chemical components used in the synthesis experimental run (Table 1).^41^ Following the scan, quantitative analysis on individual 22 britholite spots using WDS and default data reduction routines provided in the JEOL software (XPP^54^) revealed that its mass composition is 18.86 ± 0.32% SiO_2_, 1.19 ± 0.16% F, 8.12 ± 0.19% CaO, 0.07 ± 0.07% FeO, and 68.24 ± 0.50% Pr_2_O_3_, for an analytical total of 97.01 ± 0.77%. In contrast to preliminary EDS data, stoichiometry based on WDS data show that Ca+Pr+Fe equal 5 apfu whereas Si is less than 3 apfu. The *T*-site silica deficiency and low analytical totals indicate the potential incorporation of light elements, most likely H or C.

**Figure 3:**
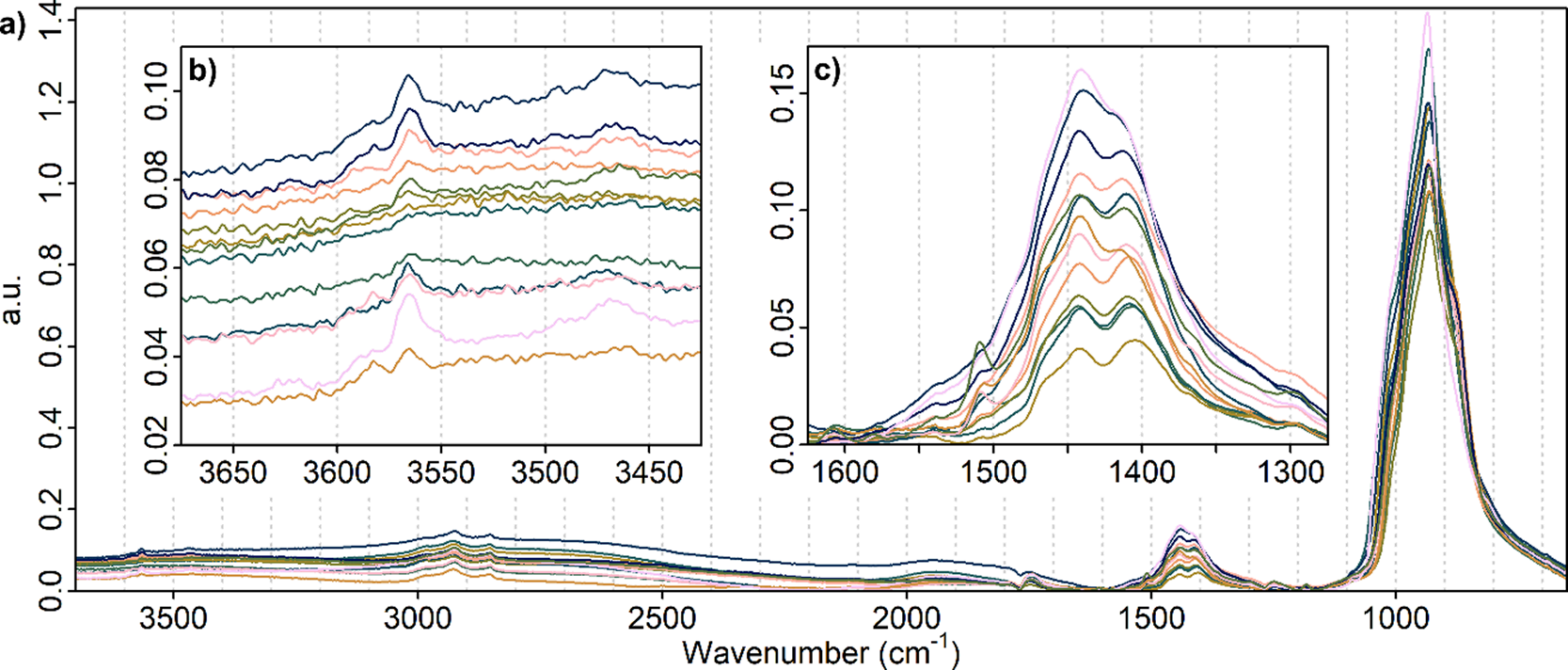
(a) Set of FTIR-ATR spectra obtained on random orientations of britholite. (b) Close-up of the H_2_O/OH^–^ region. (c) Close-up of the carbonate region.

Further investigation using FTIR showed the presence of carbonate peaks between 1400 and 1500 cm^–1^ (Figure 3),^55^ which indicate B-type carbonate substitution on the *T*-site.^56−61^ The observed B-type peaks are characteristic for apatite supergroup materials, and distinct from other carbonate-bearing minerals.^62^ FTIR also revealed minor OH contents (at around 3560 cm^–1^, Figure 3), which were estimated using the relative ratios of the OH and carbonate peaks following previous calibrations to be equivalent to 0.063% H_2_O assuming that carbonate contents fill the *T*-site to 3 apfu.^63,64^

**Figure 4:**
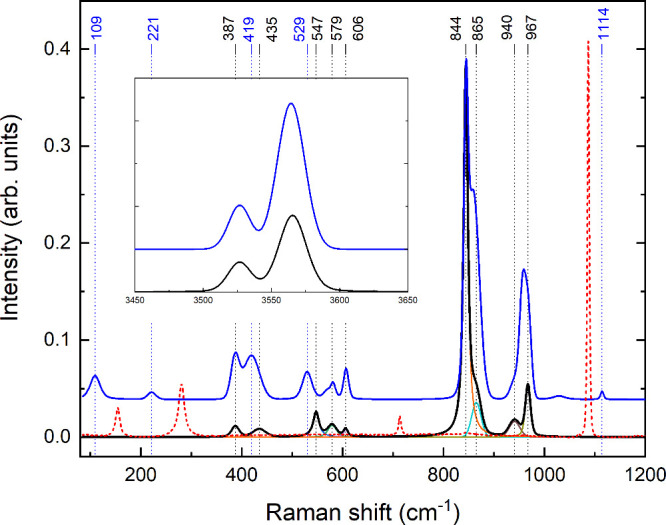
Raman spectra of two different britholite grains (black and blue curves). Red dotted curve shows Raman spectrum of britholite-associated calcite. Cyan and brown curves show deconvolution of measured data to individual peaks. Inset shows the region of O–H stretching vibration modes.

Carbonate was also confirmed by using Raman spectroscopy on different britholite grains (Figure 4). The britholite crystal structure is similar to apatite, guiding the interpretation of the britholite spectra.^65^ Raman bands at 940, 950, and 967 cm^–1^ correspond to the ν_1_ mode of symmetrical SiO_4_^4–^ tetrahedra vibrations. A distinct band at 844 cm^–1^ results from symmetric stretching of SiO_4_^4–^ groups.^66^ The bands at 387, 419, and 435 cm^–1^ are attributed to the ν_2_ mode bending vibrations of SiO_4_^4–^. The bands at 529, 547, 579, and 606 cm^–1^ are related to the ν_4_ bending mode. The bands at 109 and 221 cm^–1^ are due to the lattice modes of the britholite. The weak band at 1114 cm^–1^ corresponds to the ν_3_ asymmetrical stretching vibrations of SiO_4_^4–^ tetrahedra or the ν_1_ CO_3_^2–^ mode. The band at 865 cm^–1^ is attributed to overlapping of the ν_4_ modes of the CO_3_^2–^ and Si–OH stretching vibration. The bands at 3527 and 3565 cm^–1^ are due to the stretching vibration of O–H. The differences between the spectra of the two grains are attributed to the different orientations of the grains relative to the incident laser beam in the Raman spectrometer.

The spectrum of calcite, which is found in association with britholite, is presented in Figure 4 (dashed curve). The Raman bands of calcite differ from those of britholite, demonstrating that the britholite spectrum does not suffer from calcite contamination.

In order to improve the accuracy of light element quantification (H, C, and F) and stoichiometric determination, we attempted to recalculate the britholite composition based on the EPMA raw data output using CalcZAF (v.12.8.9, after CITZAF^67^). However, any material such as a carbonated lanthanide phosphate contains atoms of highly contrasting atomic mass, meaning that absorption within the material can be large and requires large ZAF corrections. Differences and systematic errors in correction procedures caused by the different underlying physical models can therefore result in significant differences between the true and calculated compositions. This is especially problematic where some elements must be calculated by difference, in this case, C, because that difference incorporates both analytical uncertainty and the cumulative uncertainty in the intensities of all analyzed elements. Our approach was to simulate the X-rays emitted from a hypothetical britholite with a composition close to our unknown and to quantify those X-rays as if they are an unknown material. By identifying the calculation conditions that could most successfully recover the composition of our simulated britholite, we could then proceed to quantify our real EPMA measurements of the unknown britholite with the highest accuracy.

The predicted characteristic X-ray yield was modeled with Monte Carlo simulations performed with the simulation package PENEPMA (v.2014),^68^ which is a package of the general-purpose PENELOPE code^69^ optimized for EPMA applications that has been demonstrated to be accurate.^70^ For our simulation, a hypothetical britholite was defined with a composition and density as similar as possible to those of the measured unknown britholite. A 1 cm-diameter, 1 cm-deep disk of the composition (Ca_1.4_Pr_3.6_)_5.0_(Si_2.8_C_0.2_)_3.0_O_12_(F_0.4_O_0.6_)_1.0_ with a density of 5.129 g cm^–3^ was centered perpendicular to the simulated incident electron beam. This composition was selected based on preliminary uncorrected data obtained from EPMA and assumed stoichiometry.

To simulate realistic EPMA conditions, an accelerating voltage of 15 kV was used, and generated X-rays were counted by an annular (360°) detector with a 10° opening from 35 to 45° was to simulate the typical 40° takeoff angle in the EPMA instrument. Interaction forcings were applied to increase computational efficiency.^71^ The britholite simulation was run until the relative 3σ uncertainty on the F kα intensity was reduced to around 0.04%, requiring around 24 million electrons; all other elements had a relative uncertainty better than 0.02%. A lower energy cutoff of 500 eV was applied to increase simulation efficiency. This meant that—like the real unknown measurements—C was not directly measured; rather, its concentration was considered a known parameter. Oxygen was likewise not measured but was calculated by stoichiometry. Other elements were quantified with pure standards of the same geometry that were simulated under the same conditions: diopside (Si and Ca); fluorite (F); and Pr-pentaphosphate (Pr). Simulations of standards were run until the element of interest reached a 3σ relative uncertainty of 0.02 (Pr) or 0.01 (other elements).

Just as for the real EPMA measurements, relative X-ray intensities from the simulated “unknown” (Ca_1.4_Pr_3.6_)(Si_2.8_C_0.2_)O_12_(F_0.4_O_0.6_) were quantified from k-ratios of known simulated standards using the software package CalcZAF.^72^ Using CalcZAF, we could explore the effects of using a range of typical ZAF and (φ)ρz correction procedures and different MAC tables on the accuracy of the quantification of various oxide concentrations of the britholite. We found that the Heinrich/Duncumb-Reed correction quantified all elements the best. Of all the MAC tables, LINEMU, the default in CalcZAF, performed most accurately for this particular composition.

Based on this investigation, we chose to quantify our unknown from EPMA measurements, including the C content by difference and O by stoichiometry, with iterative calculations using the Heinrich/Duncumb-Reed correction routine with LINEMU MACs. Our approach consisted of initially calculating a C-free composition, normalizing all *M*-site cations (Ca + Pr + Fe) to 5, and calculating C by difference from the Si deficit in the *T*-site. Next, the by-difference calculated C contents were fed back to CalcZAF and the composition was calculated again. This was repeated several times until the *T*-site contained 3 apfu to within 0.01 atoms. Our final result is an average of this process on 20 analytical spots, giving the composition (Ca_1.33_Pr_3.66_Fe_0.01_)_∑=5_(Si_2.83_C_0.17_)_∑=3_O_12_(F_0.58_OH_0.06_O_0.36_)_∑=1_[O_0.15_]. For charge balance, 0.15 apfu of excess O^2–^ were required, which constitute 1.19% of the total O budget of the chemical formula. Given the large number of assumptions and uncertainties inherent to this calculation, this is an excellent result and provides additional confirmation for carbonate incorporation in britholite. The excess negative charge also argues against the presence of *X*-site vacancies,^73^ as these would increase the negative charge imbalance.^74^ Analytical totals are 100.53%, which likewise indicate an excellent result, as apatite-group materials are notorious for totals that strongly differ from 100%.

### Crystal Structure

The full results of crystal structure determination are deposited in the Cambridge Crystallographic Data Centre (CCDC) under entry number 2314987. Full SCXRD data including final atom coordinates, displacement parameters and site occupancies are given in the Supporting Information (Tables S1 and S2). Selected interatomic distances are reported in Table S3 of the Supporting Information.

**Figure 5:**
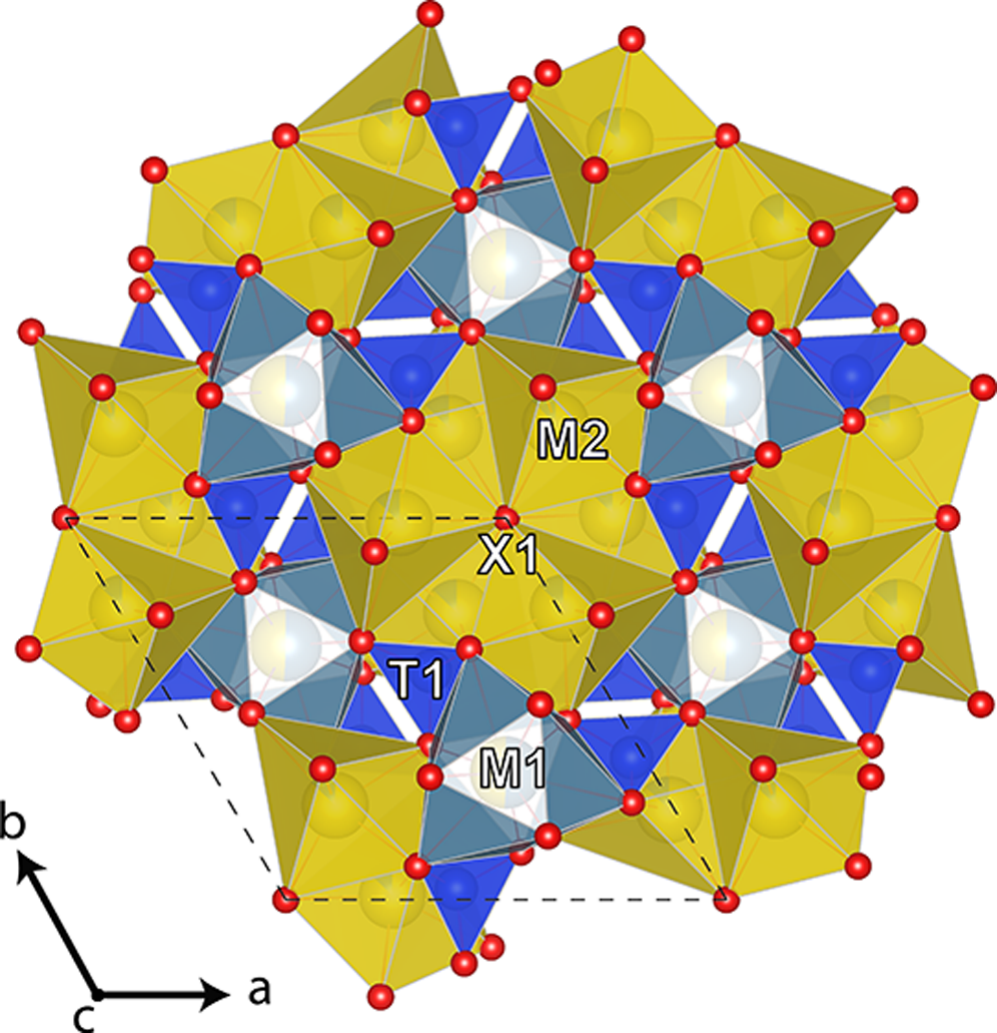
General top view of the britholite structure projected along the *c* axis. The unit cell is outlined in the dashed line. Oxygen atoms in red, *T*-site tetrahedra in blue, *M*1-site polyhedra in light gray, and *M*2-site polyhedra in yellow.

**Figure 6:**
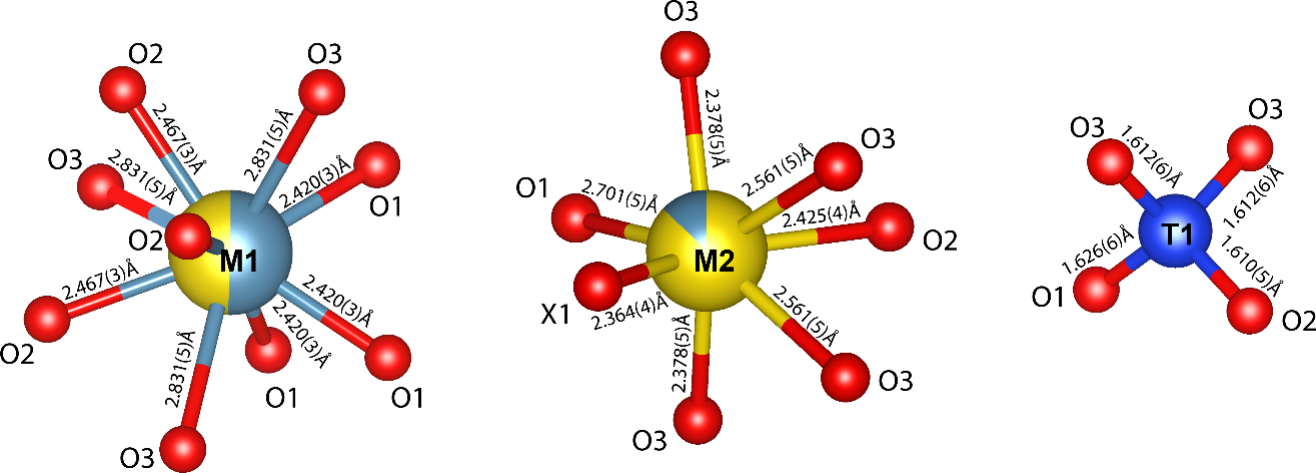
Coordination of cationic sites in the britholite crystal structure. Oxygen atoms are colored red, calcium in teal, praseodymium in yellow, and *T*-site atoms in blue.

The crystal structure of apatite-related compounds based upon heteropolyhedral framework that consists of *M*1 tricapped trigonal prism (9-coordinated) edge-shared with 7-coordinated *M*2 site and *T*O_4_ tetrahedra.^45^ The general view of our britholite structure projected along its *c* axis is shown in Figure 5. The *M*1 (4f) site is nearly equally populated by Ca and Pr, and its refined occupancy is (Ca_0.54_Pr_0.46_)_1.00_. The mean *M*1–O bond lengths of 2.573 Å are consistent with 2.523 Å in britholite-(Ce).^75^ The *M*2 (6h) site is predominately occupied by Pr with total occupancy of (Pr_0.85_Ca_0.15_)_1.00_ (Figure 6), consistent with previous studies showing lanthanide preference for the *M*2 site.^76^ The mean *T*1–O bond lengths of 1.615 Å and its scattering factor are slightly less than those of full occupancy by Si atoms only. The thermal ellipsoid of *X*1 site (see Supporting Information, Table S3) has elongation along *c* axis, a typical feature for fluorine-bearing apatite supergroup species.^73^ During refinement, the *X*1 (4e) site has split into two subsites with *X*1-*X*1 distance of 0.981 Å and total occupancy of each subsite fixed with 0.5. Such splitting may be occurring through local O–F ordering (due its near equal occupancy). The total refined occupancy of the *X*1 site is F_0.54_O_0.46_ is in a good agreement with EPMA data (neglecting minor OH^–^ contents, challenging to distinguish using XRD). Insofar as the Pr^3+^ cations are partially ordered among *M*-sites, no symmetry lowering was observed from *P*6_3_/*m* symmetry, as observed previously.^75,77^

**Figure 7:**
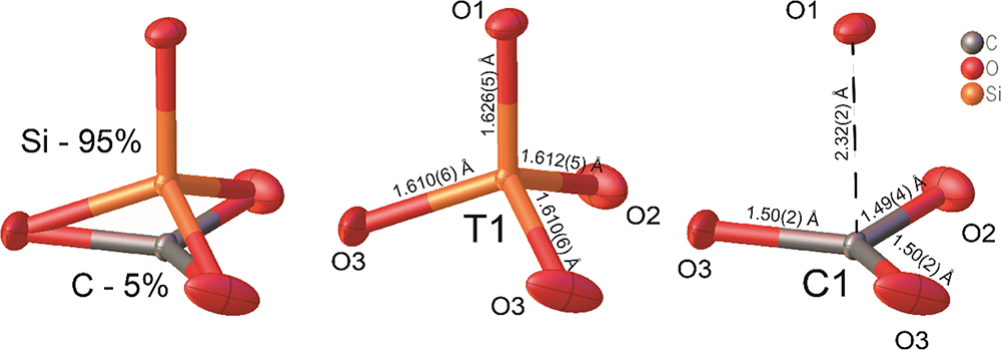
Arrangement of CO_3_ groups on *T*1 tetrahedra faces.

As a second step of the crystal structure refinement process, we attempted to find a C atom in the *T* site by using indirect parameters. The *T*–O(1) bond of 1.626 Å is elongated compared to other tetrahedral bonds (1.610 × 2 and 1.612). Together with the increase of O atom thermal ellipsoids in the triangle O(3)–O(2)–(3), we expect the carbonate anion to be present at this face. The possible presence of roughly 5% CO_3_^2–^ (i.e., 0.15 apfu) at these *T*1-tetrahedra faces is in excellent agreement with EPMA chemical determination (Figure 7). Our model explains the presence of the Si vacancy, where the *T* site is populated with C. The carbonate group has a triangular coordination, and as such it is located on the *T*1 tetrahedron face, whereas the tetrahedron center now contains a vacancy.

**Figure 8:**
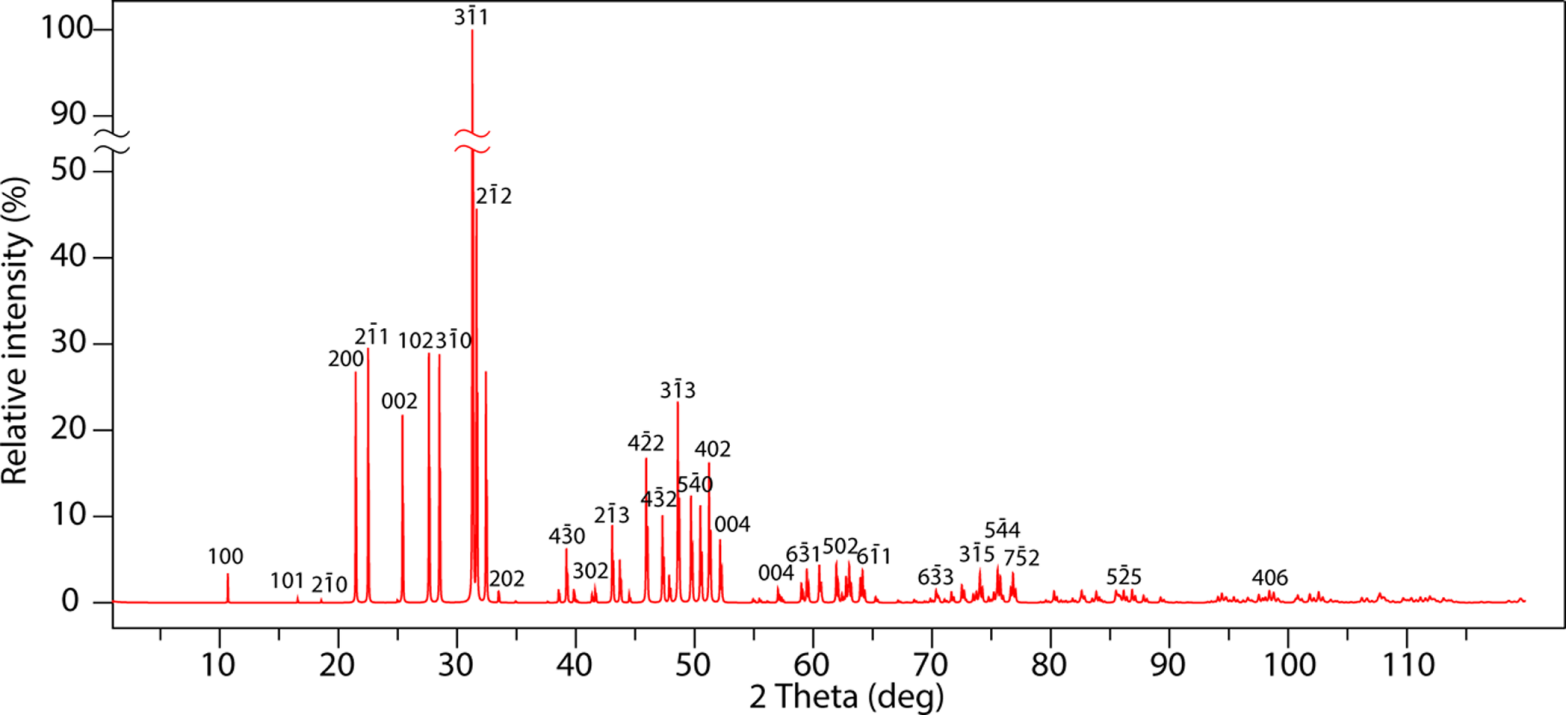
Simulated britholite powder XRD pattern using *a* = 9.5588(4) Å and *c* = 7.0097(4) Å. Calculated using VESTA.^46^

The full crystal-chemical formula for the synthesized oxybritholite, as determined by crystal structure refinement, is (Pr_3.46_Ca_1.54_)_5_(Si_2.85_C_0.15_)_3_O_12_(O_0.54_F_0.46_). Inclusion of carbonate in apatite-group minerals is occasionally explained by formation of vacancies in the *M*-site,^78−80^ but in our case, a quadrivalent cation (C^4+^) substitutes for another quadrivalent cation (Si^4+^), and the surrounding oxygens are merely structurally rearranged, thus no vacancy formation is required. A simulated powder XRD pattern derived from SCXRD data is given in Figure 8.

### DFT Optimization

**Figure 9:**
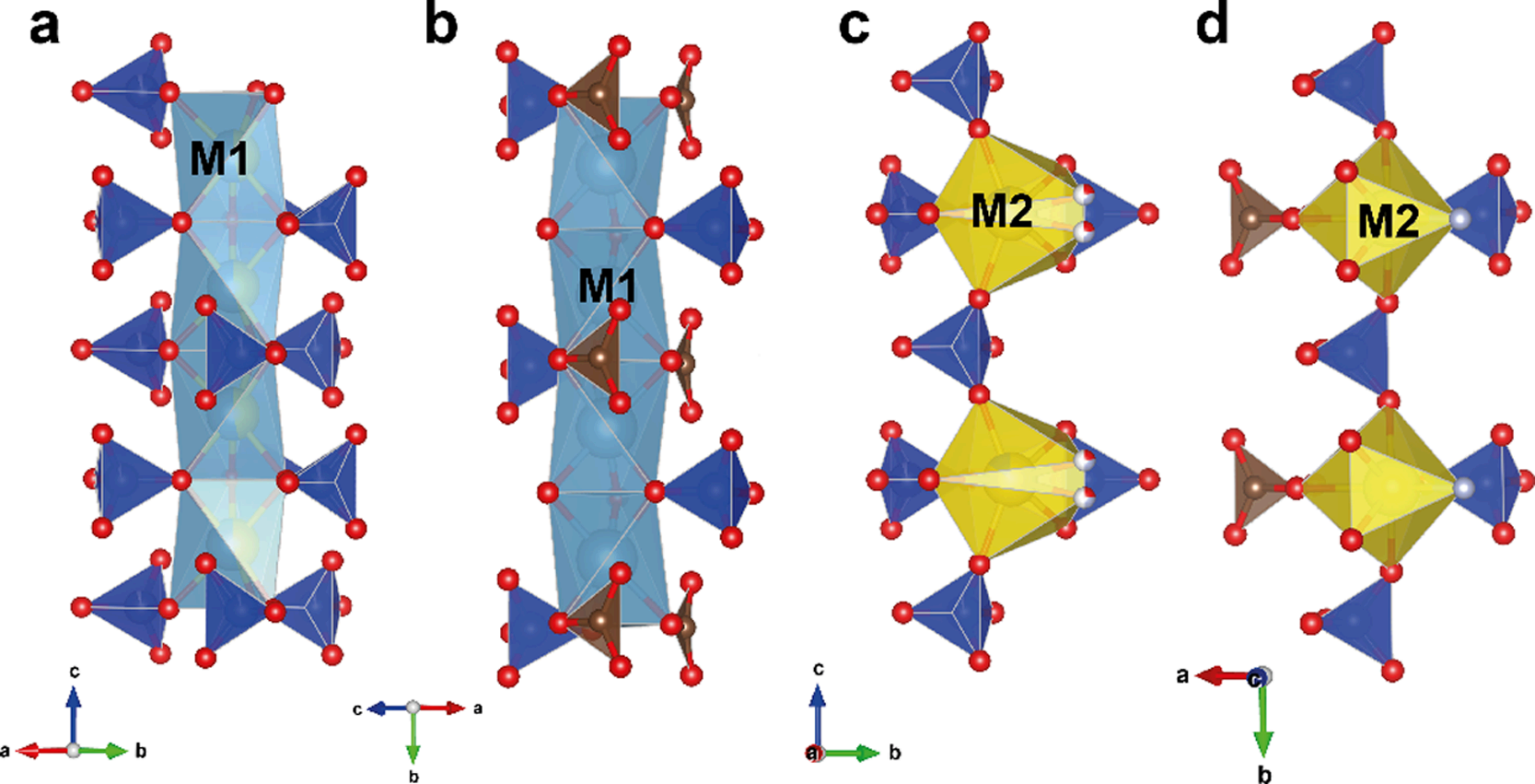
Arrangement of *M*1O6 and *M*2O6*X*1 columns in the refined (a, c) and DFT-optimized (b, d) structure.

The energy minimization procedure led to the refined parameters of the britholite unit cell of *a* = 9.3353 Å, *b* = 6.7451 Å, *c* = 9.3358, and β = 119.97°. The procedure revealed a symmetry reduction from *P*6_3_/*m* to *Pm* and a unit cell volume reduction from 554 to 509 Å^3^. We found that Δ*E* = 5.79235 eV/cell with the final energy being *E* = –24805.38262 eV/cell and the final free energy being *F* = *E*–*TS* = –24805.53929 eV/cell. Fractional coordinates of the ground state structure units are presented in the Supporting Information (Table S4). The refined formula is Ca_4_Pr_6_C_3_Si_3_O_24_F_2_. In general, the Si–O, *M*1–O, and *M*2–O distances in optimized model are consistent with the same values in known britholite structures.^75,77^ The geometry is also consistent with initial assumption, but we note slightly increased values of C–O bonds in CO_3_^2–^ triangles of 1.38–1.40 Å compared to the calculated values of 1.32 Å.^81^Figure 9 shows the fragments in the refined and DFT-optimized structure. The replacement of half of the Si by C sites leads to the loss of center of symmetry. Nevertheless, the whole structure topology remains the same.

### Photoluminescence

**Figure 10:**
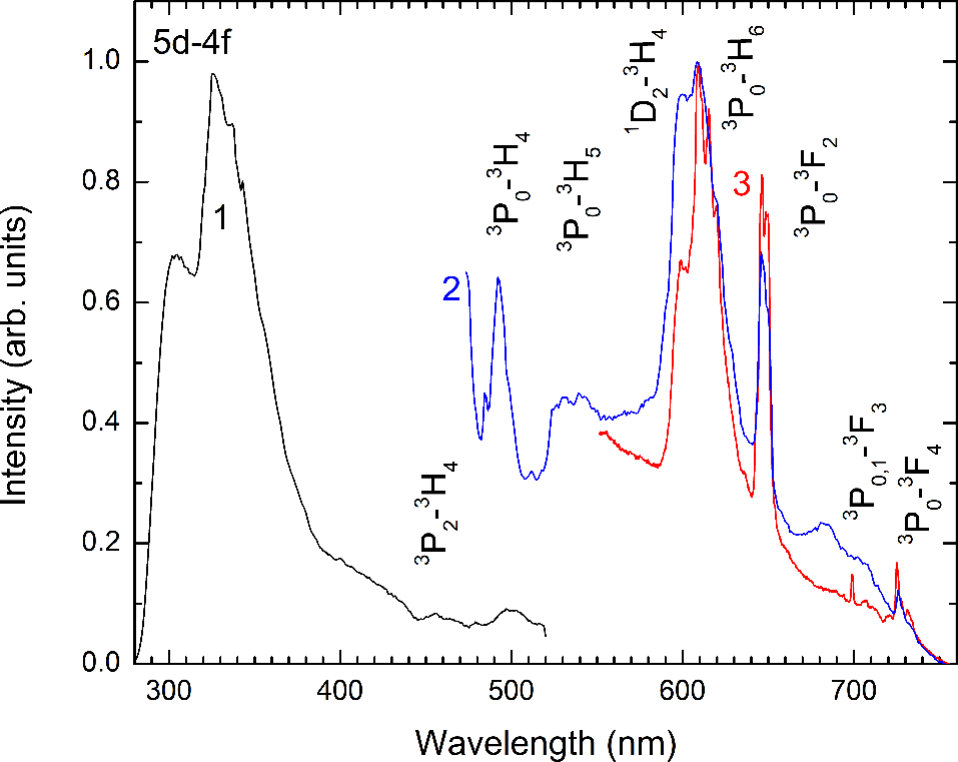
Luminescence spectra of the britholite sample under 266 nm excitation (curve 1), and 447 nm excitation in two different spots (curves 2 and 3).

The presence of CO_3_^2–^ groups would trigger local geometry of both *M*1 and *M*2 polyhedra to change and may affect luminescent properties. Therefore, we investigated the optical properties of our synthetic britholite. The photoluminescence spectrum under 266 nm excitation is shown as curve 1 in Figure 10. Two relatively wide bands with maxima at 305 and 325 nm are observed. These bands are attributed to 5d–4f transitions in Pr^3+^ ions. In silicates such as LiLa_9_(SiO_4_)_6_O_2_ with an apatite structure, the 5d–4f luminescence bands have been observed in the region of 295–344 nm for Pr^3+^ ions in low symmetry point group ligands.^82,83^

In phosphate-dominated apatite supergroup materials, the Pr^3+^ 5d–4f luminescence bands are typically found in the 240–280 nm region, whereas the bands at 305 and 325 nm correspond to 5d–4f transitions of Ce^3+^.^83^ However, in apatite-structured silicates such as LiLa_9_(SiO_4_)_6_O_2_, the 5d–4f luminescence bands have been observed at longer wavelengths or lower energy region, specifically in the range of 295–344 nm for Pr^3+^ ions in low symmetry point group ligands.^82^ On the one hand, silicate-containing complexes exhibit higher polarizability compared to phosphates, resulting in an energy shift of 4f–5d transitions to lower energy regions in silicate complexes. The presence of carbonate adjacent to Pr^3+^ cations, for the same reason, can cause an even greater shift of the band toward lower energies.^84^ On the other hand, the position of the Ce^3+^ luminescence band can be estimated to be around 410 nm based on the mean *M*2–O distance.^85^ Therefore, we infer that the observed luminescence bands at 305 and 325 nm are attributed to 5d–4f transitions in Pr^3+^ ions.

A reddish luminescence is observed in the samples under 447 nm excitation (Figure 10, curves 2 and 3). Bands at 490, 531, 600, 610, 650, 686, 709, and 727 nm are observed, as previously reported.^9^ These bands correspond to intraconfigurational 4f–4f transitions in Pr^3+^ ions. The sample contains two regions that differ in the shape of the bands at 600 and 610 nm. These regions contain a different concentration of carbonate groups as was measured by FTIR-ATR spectroscopy (Figure 3). The high-carbonate britholite crystals demonstrate widening luminescence bands due to the presence of cationic vacancies and a higher disordering of the local Pr^3+^ environment (Figure 10, curve 2). The crystals with a lower concentration of carbonate group demonstrate well-resolved luminescence bands (Figure 10, curve 3).

While the excitation energy is close to the ^3^H_4_–^1^I_6_ electron transition, the band attributed to the transition from the ^1^I_6_ and neighboring ^3^P_0_ levels with blue luminescence is located at 490 nm. The strong red luminescence at 600 nm occurs from the ^3^P_0_ to ^3^H_6_ and luminescence at 610 nm is from the ^1^D_2_ to ^3^H_4_ level due to multiphonon excitation from the ^3^P_0_ to ^1^D_2_ level at room temperature. The main phonon frequency is about 970 cm^–1^, and the distance between the ^1^D_2_ and ^3^P_0_ levels is about 3880 cm^–1^,^86^ which corresponds to four phonons. The main phonon frequency of carbonate groups that is located near Pr^3+^ is higher, and only three required phonons. Therefore, the nonradiative rate from ^3^P_0_ to ^1^D_2_ is faster than the radiative decay from the ^3^P_0_-^3^H_6_ level, and the relationship between 600 and 610 nm bands is different. The 648 nm band corresponds to ^3^P_0_-^3^F_2_ transitions, while the 680–710 nm bands are due to ^3^P_0,1_-^3^F_3_ transitions, and the bands at 727 nm are attributed to the ^3^P_0_–^3^F_4_ transition.

## Discussion

The method presented here allows growth of well-crystallized britholite grains several tens of micrometres wide. The key to reaching this size is the separation of starting materials. Instead of preparing a well-homogenized reagent mix, the chemical components are added as distinct layers to the capsule (Table 1). This retards britholite nucleation because the lanthanide layer is initially starved of the other components (e.g., CaO, SiO_2_). Crystal growth proceeds by transport via the hydrothermal fluid, in a manner similar to chemical vapor deposition, albeit at high pressure instead of vacuum.

### Carbonate in Britholite

**Figure 11:**
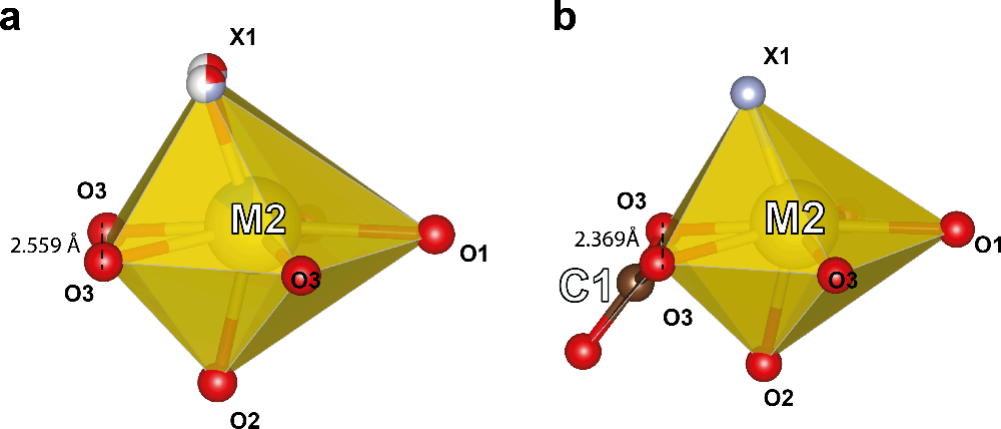
Location coordination of the *M*2 site in the crystal structure of the investigated britholite with (a) 5% occupancy of a *C*1 site, and (b) DFT model with a fully occupied *C*1 site.

In our britholite, C atoms occupy the face of the O3–O2–O3 SiO_4_ tetrahedra. Increasing Si^4+^ → C^4+^ substitution leads to the shortening of the number of O–O contacts in the carbonate triangle face. Typically, O–O distances in SiO_4_^4–^ tetrahedra of britholite range from 2.54 to 2.67 Å.^78,79^ Full occupancy of the C1 site with a mean C–O bond distance of 1.32 Å^81^ leads to a decrease of the O–O distances in the carbonate triangle face to 2.25 Å. We observe the same O–O contact length decrease in our synthetic material as well. The DFT calculations demonstrate that local symmetry changes from *C*_s_ to *C*_1_ for the *M*2 site, whereas the *M*1 site preserves *C*_3_ symmetry. Full occupancy of the C1 site leads to shortening of the O3–O3 distance from 2.559 to 2.369 Å and decrease of the O3-*M*2-O3 angle from 59.9 to 56.7° in the DFT model compared with our initial structure determination (Figure 11). Since CO_3_^2–^ triangles and SiO_4_^4–^ tetrahedra are stiffer polyhedra compared with *M*1 and *M*2, increasing the degree of occupancy of the C1 position will locally change the coordination of Pr^3+^ in the *M*2 site. This causes the gradient change in the britholite luminescence properties as a function of carbonate content, widening spectral emission bands.

Our discovery of the previously unrecognized carbonate substitution in britholite raises a concern and an application. The concern is the unintended incorporation of carbonate. Many synthesis routes include the use of organic materials or carbonates in the starting materials.^1,2,7,12,14,21−23,28,29,31,32,35,87,88^ Indeed, carbonate is occasionally observed in synthetic apatites and britholites, even though it is presumably volatile and expected to degas as CO_2_ during calcining.^1,3,13,31,89,90^ Evidently, some carbonate is stabilized and retained in the crystal lattice.^80^ As starting materials are usually prepared stoichiometrically, this leads to the problem of excess Si, which could then either precipitate as a silica polymorph (SiO_2_—either quartz or tridymite), or bond with other components in the system to form other byproduct phases (e.g., CaSiO_3_—wollastonite). This may result in impure material of inferior quality, which may not always be easily discernible, particularly when synthesizing nanoscale materials.^33^ Careful stoichiometry control by calcining of starting materials and subsequent confirmation of complete carbonate loss using FTIR is highly recommended.^91^ Furthermore, as carbon is not easily detected or analyzed using electron beam methods and is not readily obvious in XRD studies due to its low electron density, its presence might be overlooked—potentially leading to erroneous determinations of stoichiometry, composition, and inferred vacancies. However, the detection of carbonate is straightforward using FTIR, and we encourage all researchers to specifically look for the carbonate peaks.

The inclusion of carbonate via solid solution is not necessarily a nuisance but can also be exploited. As seen in the structure determination, some oxygen to metal cation bond lengths are shorter when adjacent to a carbonate group. This shortening alters the local environment and bond energies of some metal cations, but not others, leading to additional vibrational and emission bands compared to carbonate-free compounds (e.g., Figure 10).^92^ Our britholites were grown at high pressure in a hydrothermal environment saturated with carbonate (in the form of calcite), maximizing the amount of carbonate incorporated into britholite. It remains to be tested what levels of carbonate can be sequestered in solid solution when traditional solid-state sintering methods are employed, ideally in a CO_2_ atmosphere. Interestingly, a previous study concluded that carbonate promotes the introduction of lanthanides into the apatite crystal structure, in the absence of silica.^80^ Using the example of europium, they suggest that the replacement mechanism was 3Ca^2+^ = 2Eu^3+^ + □. Our results suggest that perhaps the alternative vector of Ca^2+^ + P^5+^ = Eu^3+^ + C^4+^ is responsible as a suitable pathway for the introduction of lanthanide to apatite-type materials.

### Oxybritholite

Ca–*Ln*–P–Si-apatites may be more accurately represented as an apatite–britholite–oxybritholite ternary system instead of an apatite–britholite binary system. This relation was well demonstrated in a previous study.^25^ They attempted to synthesize compositions along the hydroxylapatite–britholite–(Y) binary at 650 °C and 1.5 kbar, but found that as the Y contents increased, so did proton vacancies (i.e., OH → O + □). Evidently, the britholite and oxybritholite components in their apatite increased simultaneously.

Accurate control of OH^–^ contents has been previously demonstrated to enhance luminescent properties of apatite.^93^ The equilibrium relations governing the introduction of nonstoichiometric lanthanides into britholite or fluorbritholite via the oxybritholite component are
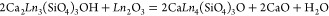

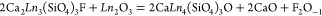
where “F_2_O_–1_” is the charge-neutral thermodynamic component of F when all components are considered as an oxide species and can be considered as the result of separating CaF_2_ into CaO and F_2_O_–1_.^94,95^ The equilibrium constants can be formulated as
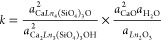

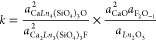
where *k* is the equilibrium constant at any combination of pressure and temperature. Thus, when grown hydrothermally, the ratio of the britholite and fluorbritholite components to the oxybritholite component (and as a corollary, the Ca/*Ln* ratio) in the desired product can be varied by controlling the H_2_O activity. For a fixed Ca/*Ln* activity ratio, the oxybritholite component would be stabilized in fluids where the F or H_2_O components has been diluted. These dilutants could be other volatile components (e.g., CO_2_, SO_2_), acids (e.g., HCl), or other species (e.g., SiO_2_, NaCl). This equilibrium occurs independently of the apatite–britholite exchange (Ca^2+^ + P^5+^ = *Ln*^3+^ + Si^4+^). In a synthetic system, it might be easier to add other components that are incompatible in britholite. Possible candidates could be heavy alkali metal halides consisting of Rb, Cs, Br and I, which together act to dilute H_2_O and control F_2_O_–1_ activity. At lower temperatures (e.g., lower than 400 °C), acid–base reactions become important, and the reaction can be rewritten as
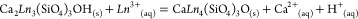


Therefore, the britholite/oxybritholite ratio in the product can also be controlled by the pH of the hydrothermal fluid, with the oxybritholite component preferred at basic conditions (high pH).

### Geological Implications

The britholites are a mineral group within the apatite supergroup^45^ with the general formula Ca_2_*Ln*_3_(SiO_4_)_3_OH (where *Ln* are the lanthanides La–Lu and Y). Currently, two species of britholite are recognized: britholite-(Ce), and britholite-(Y),^77,96,97^ with britholite-(La) described,^98^ but not formally approved by the IMA. An additional species with intermediate apatite–britholite composition, where Ca > *Ln* and Si > P, is named calciobritholite,^99^ although not formally IMA-approved. Fluorine-rich britholites are also known (where F^–^ substitutes for OH^–^) and contain the prefix “fluor” in their name: fluorbritholite-(Ce), fluorbritholite-(Y), fluorbritholite-(Nd), and fluorcalciobritholite.^66,100−102^

Currently, the two substitution mechanisms introducing *Ln* into apatite strongly supported by data are Ca^2+^ + P^5+^ = *Ln*^3+^ + Si^4+^, and 2Ca^2+^ = Na^+^ + *Ln*^3+^.^99,103−122^ However, the correlation between *Ln* and Na or Si is not always perfect.^123^ Although this mismatch can be often attributed to analytical uncertainties,^111^ our geologically reasonable conditions employed in the synthesis described above indicate that an oxybritholite component may contribute to the mismatch. Silica-deficient and low-totals britholites observed in some localities are potential hosts for undetected carbonates.^124−126^ In some cases, apatite–britholite analyses plot consistently below the 1:1 line on a Ca+P–REE+Si plot,^111,127−130^ which could be easily explained by the presence of carbonate substituting for silicate. We expect this to occur mostly in carbonatite-associated fluorapatite.^131,132^

The apatite–britholite substitution requires that endmember britholite should contain two Ca apfu and three *Ln* apfu, limited by three phosphate groups available for substitution by orthosilicate groups. However, there are reports of nonstoichiometric britholite in natural rocks containing REE/Ca > 3/2, where “Ca” includes other divalent cations that commonly substitute on the Ca-dominated *M*-site such as Sr or Mn,^75,97,101,126,133−135^ with the nonstoichiometry occasionally exacerbated by presence of phosphate (i.e., nonendmember britholite).^128^ Characterization of these natural britholites is challenging because they often contain a mix of all 14 lanthanides and other monovalent or divalent cations (e.g., Na^+^, Mn^2+^, Sr^2+^), and they may be metamict due to the presence of quadrivalent Th.^45,100,128,135^ Additionally, some orthosilicate may be substituted by phosphate or other oxyanions (carbonate, borate, arsenate, or vanadate).^136^ Finally, as britholites are apatite-supergroup minerals, they suffer the same difficulties when analyzing for the halogens F and Cl,^48−50^ and hydroxyl analysis requires separate methods (e.g., SIMS or FTIR). Therefore, establishing the precise stoichiometry of a britholite^137^ in order to understand the crystal chemical constraints that allow incorporation of the excess lanthanides is fraught with uncertainties. This has led to a plethora of proposed substitution mechanisms, but evidence for one mechanism or the other has hitherto been inconclusive.^74^

The data presented here provide strong support for the stability of the oxybritholite component under geologically reasonable conditions. In contrast, we found no evidence for excess lanthanide incorporation into britholite by vacancy as suggested by some authors (e.g., 3Ca^2+^ = 2*Ln*^3+^ + □).^138,139^ Endmember or near-endmember oxybritholite has not yet been found in natural rocks. In our case, the studied britholite crystals were homogeneous, large enough for single crystal diffraction, and were dominated by the oxybritholite component. Unfortunately, natural apatite supergroup minerals are not as simple,^140^ and it remains to be seen whether an oxybritholite component can be detected in future studies. However, an appreciable amount of excess lanthanides in britholite is occasionally found. For example, lanthanide-rich britholites in the Norberg District, Sweden contain up to 3.55 apfu *Ln*,^133^ and an “oxy” component has been calculated for britholites from Keivy, Russia.^130^ This suggests that, pending full and accurate chemical characterization, they may be reclassified as the type locality of naturally occurring oxybritholite.

## Conclusions

We find two new substitution mechanisms that operate in apatite supergroup minerals. The first is the incorporation of additional lanthanides into the structure by formation of an oxybritholite or oxyapatite structure:
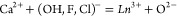


Although well-known from materials science,^8^ it has received essentially no consideration in the mineralogical literature. The endmembers formulas for oxybritholite and oxyapatite are Ca*Ln*_4_(SiO_4_)_3_O and Ca_4_*Ln*(PO_4_)_3_O, respectively. The latter is a novel substitution vector for natural apatites.

The second is the charge balanced replacement of an orthosilicate group by a carbonate and an additional oxygen:
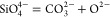


This substitution vector is currently undescribed for both natural and synthetic materials, and here, we provided the first full characterization of its structure and demonstration of its thermodynamic stability and existence. It expands the well-known “britholite component” of lanthanide incorporation in apatite (i.e., Ca^2+^ + P^5+^ = *Ln*^3+^ + Si^4+^) into an additional novel substitution vector for natural apatites:
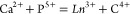


Both substitution vectors lead to increased variety in local environments and bond lengths for metal cations in the *M*1 and *M*2 sites and importantly any lanthanides. This leads to additional or wider optical emission peaks upon photoluminescent excitation.

## Supporting Information Available

Atom coordinates and equivalent displacement parameters (*U*_EQ_, Å^2^) and site occupancies in the britholite structure, nisotropic displacement parameters (Å^2^), selected bond distances (Å) in the britholite crystal structure, and atomic coordinates for the DFT-optimized structure (PDF)
