# Supplementary material for: An Apatite-Group Praseodymium Carbonate Fluoroxybritholite: Hydrothermal Synthesis, Crystal Structure, and Implications for Natural and Synthetic Britholites
Source: Inorg Chem. 2024 Jun 13;63(25):11788–801. doi: 10.1021/acs.inorgchem.4c01490 (PMC11200260; doi:10.1021/acs.inorgchem.4c01490)
Supplement: Supplementary file 1 — ic4c01490_si_001.pdf [file ic4c01490_si_001.pdf]

Supporting Information

An apatite-group praseodymium carbonate fluoroxybritholite: hydrothermal synthesis, crystal structure, and implications for natural and synthetic britholites

Michael Anenburg[1]*, Taras L. Panikorovskii[2], Eleanor S. Jennings[3]*, Roman Yu. Shendrik[4], Andrey A. Antonov[2], Veronika Gavrilenko[4]

[1] Research School of Earth Sciences, Australian National University, 8 Canberra 2600, Australia

[2] Laboratory of Nature-Inspired Technologies and Environmental Safety of the Arctic, Kola Science Centre, Russian Academy of Sciences, 184200 Apatity, Russia

[3] School of Natural Sciences, Birkbeck, University of London, London WC1E 7HX, United Kingdom

[4] Vinogradov Institute of Geochemistry, Siberian Branch, Russian Academy of 15 Sciences, 664033 Irkutsk, Russia

*Corresponding authors: michael.anenburg@anu.edu.au and e.jennings@bbk.ac.uk

Table S1. Atom coordinates and equivalent displacement parameters (U_{EQ}, Å^2) and site occupancies in the britholite structure.

Site	Occupancy	*x*	*y*	*z*	U_{eq} (Å^2)	*Wyckoff site*
*M*1	$Ca_{0.54}Pr_{0.46}$	⅔	⅓	0.50283(12)	0.0120(3)	4*f*
*M*2	$Pr_{0.85}Ca_{0.15}$	0.76410(5)	0.75257(5)	¼	0.0113(2)	6*h*
*T*1	$Si_{0.95}$	0.3727(2)	0.4014(2)	¼	0.0099(6)	6*h*
*C*1	$C_{0.05}$	0.322(4)	0.434(5)	¼	0.0082(6)	6*h*
O1	O	0.4886(7)	0.3220(7)	¼	0.0164(13)	6*h*
O2	O	0.4716(7)	0.5961(7)	¼	0.0240(16)	6*h*
O3	O	0.2546(6)	0.3427(8)	0.4332(7)	0.0358(15)	12*i*
*X*1	$F_{0.54}O_{0.46}$	0	0	0.3200	0.053(4)	4*e*

Table S2. Anisotropic displacement parameters (Å^2) for britholite.

Site	U_{11}	U_{22}	U_{33}	U_{23}	U_{13}	U_{12}
*M*1	0.0141(4)	0.0141(4)	0.0079(5)	0.000	0.000	0.00703(18)
*M*2	0.0150(3)	0.0139(3)	0.0067(3)	0.000	0.000	0.0083(2)
*T*1	0.0095(10)	0.0112(11)	0.0046(11)	0.000	0.000	0.0057(9)
C*1*	0.0095(10)	0.0112(11)	0.0046(11)	0.000	0.000	0.0057(9)
O1	0.020(3)	0.029(3)	0.012(3)	0.000	0.000	0.019(3)
O2	0.017(3)	0.016(3)	0.039(4)	0.000	0.000	0.006(2)
O3	0.029(3)	0.075(4)	0.019(3)	0.028(3)	0.013(2)	0.035(3)
*X*1	0.050(4)	0.050(4)	0.060(6)	0.000	0.000	0.025(2)

Table S3. Selected bond distances (Å) in the britholite crystal structure.

Bond	Bond distance (Å)	Bond	Bond distance (Å)
*M*1–O1 x3	2.420(4)	<*M*2–O>	2.481
*M*1–O2 x3	2.467(4)		
*M*1–O3 x3	2.831(6)	*T*1–O1	1.626(5)
<*M*1–O>	2.573	*T*1–O2	1.610(6)
		*T*1–O3 x2	1.612(5)
*M*2–O1	2.701(6)	<*T*1–O>	1.615
*M*2–O2	2.425(5)		
*M*2–O3 x2	2.378(5)	C1-O3 x2	1.50(2)
*M*2–O3 x2	2.561(5)	C1-O2	1.49(4)
*M*2–*X*1	2.364(4)	<C1–O>	1.497

Table S4. Atomic coordinates for the DFT-optimised structure.

Atom	Label	*x/a*	*y/b*	*z/c*
C:1	1	0.674811	0.500000	0.105240
C:1	2	0.570391	1.000000	0.674804
C:1	3	0.105981	1.000000	0.431059
O:1	1	0.341788	0.684210	0.253294
O:1	2	0.911780	0.683898	0.658327
O:1	3	0.743808	0.675367	0.090273
O:1	4	0.653229	1.175392	0.743828
O:1	5	0.090250	1.176234	0.346351

O:1	6	0.253389	1.184239	0.911341
O:1	7	0.341788	-0.684210	0.253294
O:1	8	0.911780	-0.683898	0.658327
O:1	9	0.743808	-0.675367	0.090273
O:1	10	0.653229	-1.175392	0.743828
O:1	11	0.090250	-1.176234	0.346351
O:1	12	0.253389	-1.184239	0.911341
O:1	13	0.321395	0.500000	0.488699
O:1	14	0.597609	0.500000	0.471998
O:1	15	1.167118	0.500000	0.677761
O:1	16	0.874476	0.500000	0.402226
O:1	17	0.509675	0.500000	-0.169174
O:1	18	0.535426	0.500000	0.125935
O:1	19	0.678335	1.000000	0.510058
O:1	20	0.409491	1.000000	0.535403
O:1	21	-0.168743	1.000000	0.320767
O:1	22	0.125912	1.000000	0.590857
O:1	23	0.488413	1.000000	1.166541
O:1	24	0.471955	1.000000	0.874073
F:1	1	0.000022	0.500000	0.000159
F:1	2	0.000044	1.000000	0.000101
Si:1	1	0.401251	0.500000	0.373742
Si:1	2	0.972387	0.500000	0.599523
Si:1	3	0.373586	1.000000	0.973145
Ca:1	1	0.334628	0.748866	0.668478
Ca:1	2	0.668047	0.247530	0.335226
Ca:1	3	0.334628	-0.748866	0.668478
Ca:1	4	0.668047	-0.247530	0.335226
Ca:2	1	0.749700	0.500000	0.762351
Ca:2	2	0.011877	0.500000	0.249470
Ca:2	3	0.237565	0.500000	0.987849
Ca:2	4	0.249970	1.000000	0.237219
Ca:2	5	0.986110	1.000000	0.748979

Ca:2	6	0.763020	1.000000	0.013804
Pr:1	1	0.334628	0.748866	0.668478
Pr:1	2	0.668047	0.247530	0.335226
Pr:1	3	0.334628	-0.748866	0.668478
Pr:1	4	0.668047	-0.247530	0.335226
Pr:2	1	0.749700	0.500000	0.762351
Pr:2	2	0.011877	0.500000	0.249470
Pr:2	3	0.237565	0.500000	0.987849
Pr:2	4	0.249970	1.000000	0.237219
Pr:2	5	0.986110	1.000000	0.748979
Pr:2	6	0.763020	1.000000	0.013804
